# Extreme low temperature is the key meteorological limiting factor for winter wheat yield in Shihezi

**DOI:** 10.3389/fpls.2026.1797185

**Published:** 2026-05-08

**Authors:** Shuai Zhang, Haonan Hu, Jingting Peng, Jianrong Zhai, Mingfeng Yang

**Affiliations:** 1 Wulanwusu Agrometeorological Experiment Station, Shawan, Tacheng Prefecture, Xinjiang Uygur Autonomous Region, China; 2 Key Laboratory of Agricultural Meteorology, Shihezi City, Eighth Division, Shawan, Tacheng Prefecture, Xinjiang Uygur Autonomous Region, China; 3 Wulanwusu Special Test Field base of National Integrated Meteorological Observation, Wulanwusu Ecology and Agrometeorology Observation and Research Station of Xinjiang, Shawan, Tacheng Prefecture, Xinjiang Uygur Autonomous Region, China; 4 College of Agriculture, Shihezi University, Shihezi, Xinjiang Uygur Autonomous Region, China

**Keywords:** extreme climate, extreme low temperature, long sequence data, winter wheat yield, yield loss ratio

## Abstract

**Introduction:**

In the background of global climate change, extreme weather events occur frequently, leading to various agricultural meteorological disasters worldwide. However, the key meteorological limiting factor for winter wheat (*Triticum aestivum* L.) production in Shihezi (Xingjiang, China) remains unclear. Clarifying this factor is a core prerequisite for ensuring regional food security.

**Methods:**

In this study, standard anomaly (s) was used to define extreme climate conditions (extreme: ≤ -2.0 s or ≥ 2.0 s; moderate: -2.0 s ~ 2.0 s).

**Results:**

Based on long-term series (1980– 2024) winter wheat yield data and daily meteorological datasets (precipitation, sunshine hours, average temperature, average maximum temperature, average minimum temperature, average wind speed) from the Wulanwusu Agricultural Meteorological Experimental Station, we quantified the impact of each meteorological factor on yield using the yield loss ratio (YLR). The results showed that extreme low temperature (average minimum temperature ≤ -2 s) is the key limiting meteorological factor for winter wheat yield in Shihezi, causing a yield reduction of 43.58%, significantly higher than the impacts of extreme wet (-12.60%) and extreme high temperature (-13.54%). Moreover, extreme low temperature mainly exacerbates yield loss by inhibiting 1000-grain weight during the tillering stages (r=-0.302).

**Discussion:**

This study identified the core meteorological limiting factors and their mechanisms for winter wheat yield in Shihezi, providing a scientific basis for optimizing stress-resistant and stablecultivation techniques and formulating climate-adaptive management strategies for regional winter wheat production.

## 1 Introduction

In the context of the sustained global warming, the frequency, intensity, and spatial scope of extreme weather events such as droughts, heat waves, storms, floods, pests and diseases have significantly increased, which will directly or indirectly damage crop productivity and exacerbate global food supply pressures (Verma et al., 2025; Kodra et al., 2011; Sanghera et al., 2011; Hasegawa et al., 2021; Ortiz-nobea et al., 2021; Augspurger, 2013). Studies have confireded that climate warming will further intensify regional climate differentiation, the degree of areas suffering from drought may further deepen, and the extreme characteristics of areas suffering from excessive rainfall may more prominent (Allen and Ingram, 2002, Allen et al., 2007, 2010; Chou et al., 2009, 2013; Li et al., 2021). Meanwhile, the rapidly growing global population has expanded food demand, creating a sharp contradiction with limited arable land resources and yield fluctuations caused by climate change (Richter and Semenov, 2004). Therefore, quantitative assessment of climate change impact on crop production is of great significance to ensure stable food supply (Feng et al., 2023; Cheng and Yin, 2022; Ali et al., 2017; Foley et al., 2005). A large number of studies have shown that the climate extreme related to temperature and precipitation, such as heat, cold and drought could severely reduce crop production from regional to global scale (Iizumi et al., 2014, 2016; Tack et al., 2015; Chen et al., 2018; Lobell and Gourdji, 2012). Extreme temperatures reduce yield by affecting crop photosynthesis, respiratory metabolism and reproductive growth (Han et al., 2025; Zhang J. B. et al., 2019), while drought suppresses nutrient absorption and material accumulation through water stress (Bogenreuther et al., 2025; Mi et al., 2025; Liu et al., 2020). However, existing research on extreme climates has mostly focused on the effects of drought (Jin et al., 2016; Ji et al., 2024) on crop production, with significant shortcomings in the systematic quantitative evaluation of extreme temperatures (especially the synergistic effects of high and low temperatures) on staple crops in specific regions.

Winter wheat is one of the three major food crops globally and a core crop ensuring China’s food security. China feeds 20% of the world’s population with 7% of its arable land, and the stability of food production is directly related to national strategic security (The State Council Information Office of the People’s Republic of China, 2019). In addition, official statistical data shows that drought, hail, wind, cold and waterlogging are the main agro-meteorological disasters restricting agricultural production in China, especially in the major winter wheat-producing areas such as the northwest, where meteorological disasters have a significant impact (National Bureau of Statistics of China, 2025. http://www.stats.gov.cn/). Xin jiang is a high-quality wheat production base in China, and its yield is crucial for regional and national food security (Chen et al., 2025). The region has a poorly stable climate system with significant interannual and intra-annual fluctuations. Wind, hail and low temperature disasters are the main meteorological factors affecting the growth, development and yield formation of winter wheat (Zhang et al., 2024). Shihezi, located on the northern slope of the Tianshan Mountains in Xinjiang, is a typical winter wheat-producing area in the region. Although previous studies have explored the effects of meteorological factors on winter wheat yield in Xinjiang, a critical knowledge gap remains: which factor is the key meteorological limiting factor in Shihezi, and to what extent do precipitation, sunshine hours, temperature, and wind speed contribute to yield changes? These gaps hinder our ability to understand and evaluate the impacts of climate change on local crop production.

To address this gap, this study analyzed the correlation between long-term meteorological data and winter wheat yield data in Shihezi, quantified the impact intensity of different extreme climate factors on yield, and analyzed the key meteorological limiting factors of winter wheat yield in Shihezi and the phased mechanism of yield reduction caused by these factors. The results can provide practical references for formulating stress-resistant and stable-yield cultivation techniques.

## 2 Materials and methods

### 2.1 Study area

**Figure 1:**
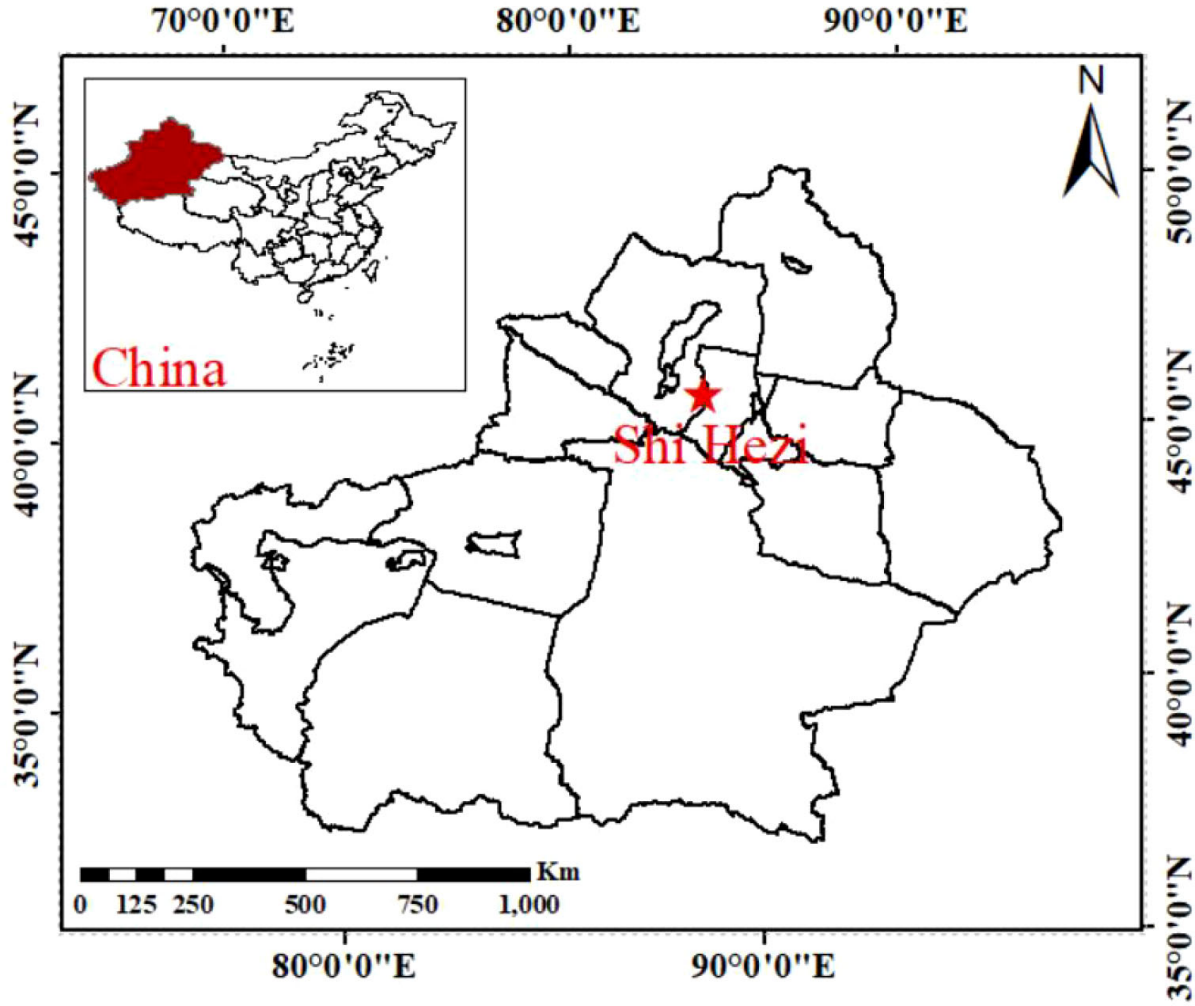
Location of study area.

The study area is Shihezi City (44°19′N–45°08′N, 85°10′E–86°24′E), located on the northern slope of the Tianshan Mountains in the economic zone of northern Xinjiang (**Figure 1**). The region has a typical temperate continental climate, with diurnal large temperature differences, long sunshine hours, and uneven precipitation distribution. Generally, winter wheat is sown in early October, overwintering from late November to early March, and is harvested around the end of June. The main growth stages include tillering (Octobe-November), jointing (March-April), heading (April-May), flowering (May) and maturity (June) (Liu et al., 2019).

### 2.2 Data source

#### 2.2.1 Meteorological data

The meteorological data used in this study were daily observations from the Wulanwusu Agricultural Meteorological Experimental Station during the period 1980-2024, including precipitation, sunshine hours, maximum temperature, minimum temperature, average temperature and wind speed. The sunshine hours fluctuated between 2031.1-3220.7 h, while annual average temperature varied between a minimum of 4.50°C (recorded in 2018) and a maximum of 10.37°C (observed in 2024). The annual precipitation varied in the range of 117.3-368.5 mm. These meteorological data effectively reflect changes in local climatic conditions over the years.

#### 2.2.2 Winter wheat yield data

Winter wheat field statistical data were also obtained from the Wulanwusu Agricultural Meteorological Experimental Station from 1980 to 2024. The highest yield reached 9.26 t hm^^-^²^ (2024) and the lowest was 3.48 t hm^-^² (1988).

### 2.3 Statistical analysis

All statistical analyses were performed using R 4.3.1 and SPSS 26.0. Graphs were generated using ArcMap 10.8 and Origin 2021.

#### 2.3.1 Definition of extreme climate condition

Standard anomaly (M, Equation 1) was used to quantify the deviation of annual climate conditions from the long-term average, as it eliminates the influence of units and magnitude differences between factors (Liu et al., 2022). A standardized anomaly below -2.0 σ or above 2.0 σ was defined as an extreme anomaly. Moderate anomalies were defined as those between -2.0 σ and 2.0 σ.

(1) $$M=\frac{Ci\;-C}{\sigma }$$

*M* = Standard anomaly of the meteorological factor;

*Ci* = Annual value of the meteorological factor (including total precipitation, sunshine hours, average temperature, average maximum temperature, average minimum temperature and average wind speed during the growing season);

*C* = Long-term average value of the factor (1980–2024);

*σ* = Standard deviation of the factor (1980–2024).

To test the robustness of the ±2σ threshold, we conducted a sensitivity analysis using alternative definitions of extremes: the 5th/95th percentiles and the 10th/90th percentiles. The rank order of factor impacts (based on YLR) was compared across these definitions to ensure that the identification of the key limiting factor was not threshold-dependent.

#### 2.3.2 Yield loss ratio

The impact of climate extremes on yield could be quantified by comparing observed yield in different year with the expected yield from the long-term trend of yield change. More specifically, it is to calculate the yield loss rate (YLR, Equation 2) by dividing the difference between the observed yield (*yield_obe_*) and the expected yield (*yield_tre_*) by the expected yield, which could reflect the deviation of yield in extreme climate years to the long-term yield trend.

(2) $$YLR=\frac{yield_{obs}-yield_{tre}}{yield_{tre}}\times 100\%$$

## 3 Results and analysis

### 3.1 Trend of winter wheat yield

**Figure 2:**
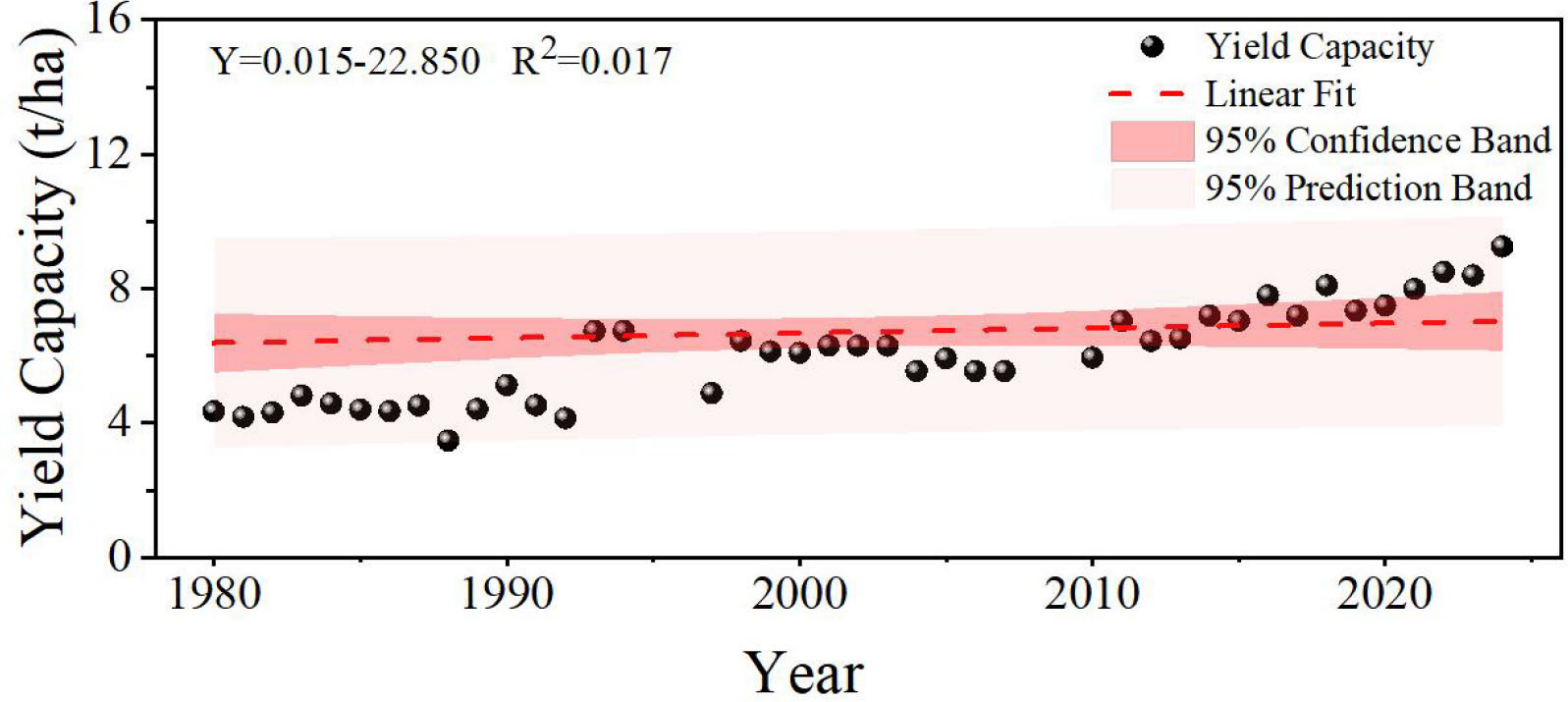
Winter wheat yield trend.

Trend analysis indicated that winter wheat yield in Shihezi has been consistently increasing from 1980 to 2024 (**Figure 2**). The LOESS smoother effectively captured the nonlinear trend, with a coefficient of determination (R²) of 0.808. Residual diagnostics showed no significant autocorrelation or heteroskedasticity, indicating that the trend was adequately removed. Meanwhile, the winter wheat yield fluctuated significantly around the fitting trend line, with particularly prominent fluctuations during the periods 1988–1998 and 2010-2020, reflecting the strong interference of complex factors such as climate variability, management measures and lags in technological innovation on yield capacity, long term linear patterns were masked by short-term fluctuations.

### 3.2 Variability of climate factors

**Figure 3:**
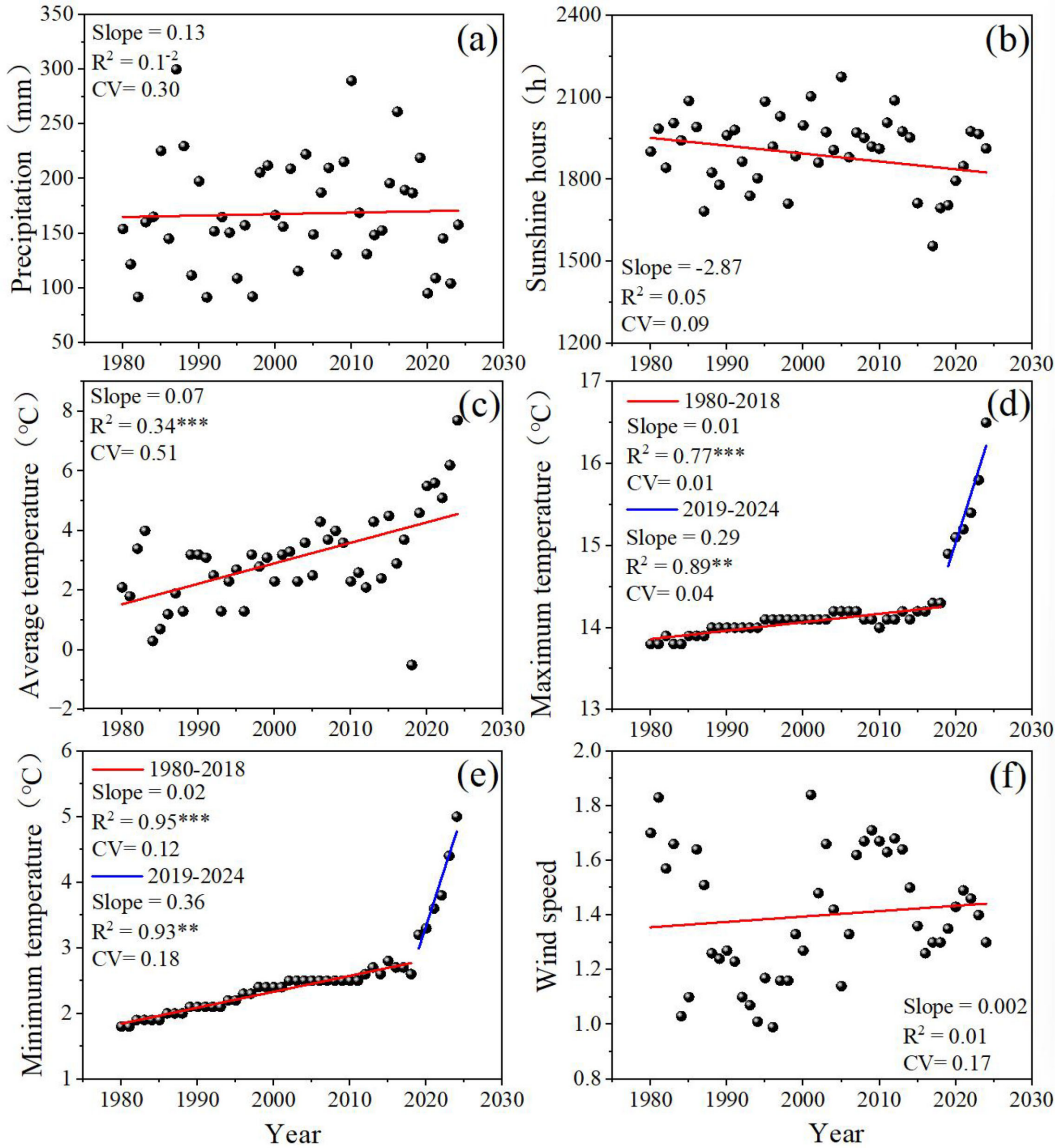
The trends and CV of total precipitation **(A)**, sunshine hours **(B)**, average temperature **(C)**, average maximum temperature **(D)**, average minimum temperature **(E)** and wind speed **(F)** during the growing season. The asterisk (*) indicates that the significant correlation.

The variability of total precipitation, mean sunshine hours, average temperature, average maximum temperature, average minimum temperature and average wind speed across the growing season of winter wheat were calculated. The spatial pattern of the coefficient of variation (CV) for different meteorological factors is shown in **Figure 3**. We could see that the CV of annual precipitation (0.30) and average temperature (0.51) across the winter wheat growing season was significantly higher, indicating that severe interannual fluctuations and poor stability of these two factors. The CVs of average minimum temperature (0.12, 0.18) and average wind speed (0.17), respectively, fell within the moderate variation range. In contrast, the CVs of average maximum temperature (0.01, 0.04), respectively, were all below 0.1, indicating that their interannual variations are stable and highly stable. Furthermore, during the winter wheat growing season, average temperature, average maximum temperature, and average minimum temperature exhibited significant increasing trends with years (**Figures 3C–E**). No significant correlations were observed between precipitation, mean sunshine hours, average wind speed and year (**Figures 3A, B, F**).

### 3.3 The impact of extreme climate on yield

**Figure 4:**
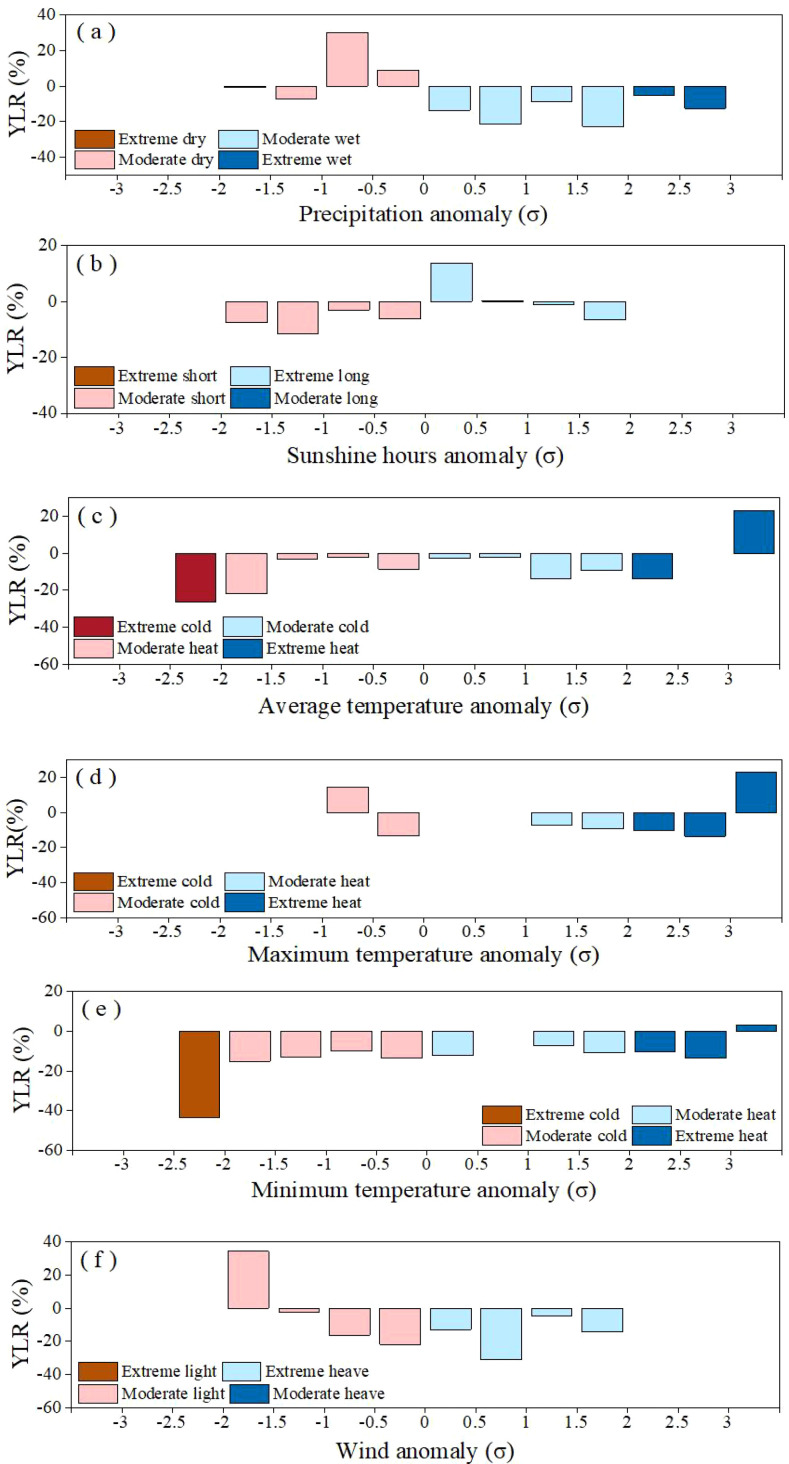
Impact of extreme climate factors, including precipitation **(A)**, sunshine hours **(B)**, average temperature **(C)**, maximum temperature **(D)**, minimum temperature **(E)** and wind **(F)** on winter wheat yield of Shihezi. YLR, yield loss ratio.

**Figure 4** shows the response of winter wheat yield (YLR) to gradients of climate anomalies, represented by precipitation, sunshine hours, maximum temperature, average minimum temperature and average wind, ranging from normal to extreme conditions over the decades in the Shihezi. The climate factor anomalies falling into the extreme intervals (≥ 2.0 σ or ≤ 2.0 σ), reflecting the impact of extreme climate. Similarly, the impact of climate anomaly gradient with an interval of 0.5 σ on yield were also obtained. The results show that positive YLR was observed when precipitation anomaly ranged from -1.0 σ to 0 σ, which means the favorable precipitation condition for winter wheat in our study area is slightly drier than the multi-year average (**Figure 4A**). When the precipitation anomaly (σ) is less than -1 σ, YLR increases significantly. However, as the precipitation condition deviates from normal to extremely wet, the reduction in yield sharply increase (5.16%~22.67%), indicating that excessive rainfall could lead to a significant decrease in yield.

Further analysis of sunshine hours anomaly (**Figure 4B**) shows that short sunshine hours could result in more yield loss than in longer sunshine hours. As the sunshine hours anomaly gradually decreases from 0 σ to −2.0 σ, the YLR gradually decreases. On the other hand, the results show that positive YLR was observed when sunshine hours anomaly ranged from 0 σ to -1.0 σ. When the sunshine hours anomaly gradually increases beyond 1 σ, the YLR gradually decreases, but the minimum does not exceed -7%. Additionally, both indicate a loss in production when sunshine hours anomaly is less -2.0 σ and greater 2.0 σ,

As for the average temperature (**Figure 4C**), both extreme heat and extreme cold could inhibit winter wheat yield, with a YLR of -26.31% and -13.54%, respectively. In addition, as the average temperature anomaly gradually increases from -2.0 σ to -0.5 σ, the YLR showed a progressive upward trend, indicating that the inhibitory effect of moderately low temperature on winter wheat yield weakened with the increase of temperature. The results also revealed that the YLR gradually increased as the average temperature anomaly ranged from 0 σ to 2.0 σ.

**Figure 4D** shows that YLR is positive when the average maximum temperature anomaly is less than -0.5, indicating that a moderate low-temperature environment may promote yield formation by prolonging crop growth period, optimizing the accumulation of photosynthetic products and other mechanisms. Additionally, YLR gradually increases (7.12%~10.31%) when the average maximum temperature falls within the moderate heat interval (0 σ ~ 2.0 σ). When the maximum temperature anomaly transforms from the moderate heat to the extreme heat (1.5 σ ~ 2.0 σ), winter wheat yield loss is, on average, -9.12%.

Analysis of average minimum temperature anomaly (**Figure 4E**) shows the winter wheat yield loss is the highest (YLR=-43.58%) when the minimum temperature anomaly is less than -2.0 σ, indicating that extreme cold is the key factor leading to a significant decrease in yield. YLR shows a downward trend when the minimum temperature anomaly increases from -2.0 σ to -0.5 σ. And when the minimum temperature anomaly is in transition from the moderate heat to the extreme heat (1.5 σ ~ 2 σ), the reduction in production increases.

The results (**Figure 4F**) indicate that impact of average wind speed on yield exhibits significant asymmetry. Positive YLR was observed when average wind speed anomaly ranged from -2.0 σ to -1.5 σ. As the average wind anomaly is in the range of -1 σ to 2 σ, it generally leads to a reduction in production, with the greatest reduction occurring when the wind anomaly is greater 0.5 σ, at -31.23%. Moreover, both indicate a loss in production when the wind anomaly is less than -2.0 σ and greater 2.0 σ, indicating that extreme strong wind weather has not occurred in the Shihezi area. The sensitivity analysis using alternative thresholds (5th/95th and 10th/90th percentiles) confirmed that extreme low temperature consistently ranked as the most damaging factor (**Table 1**).

**Table 1:** Sensitivity analysis of YLR for extreme climate events under different threshold definitions.

Climate factor	Extreme event	YLR (± 2σ)	YLR (5th/95th)	YLR (10th/90th)
Min Temperature	Cold Extreme	-43.6%	-41.2%	-38.5%
Precipitation	Wet Extreme	-12.6%	-11.8%	-9.9%
Avg Temperature	Hot Extreme	-135%	-12.1%	-10.8%

**Figure 5:**
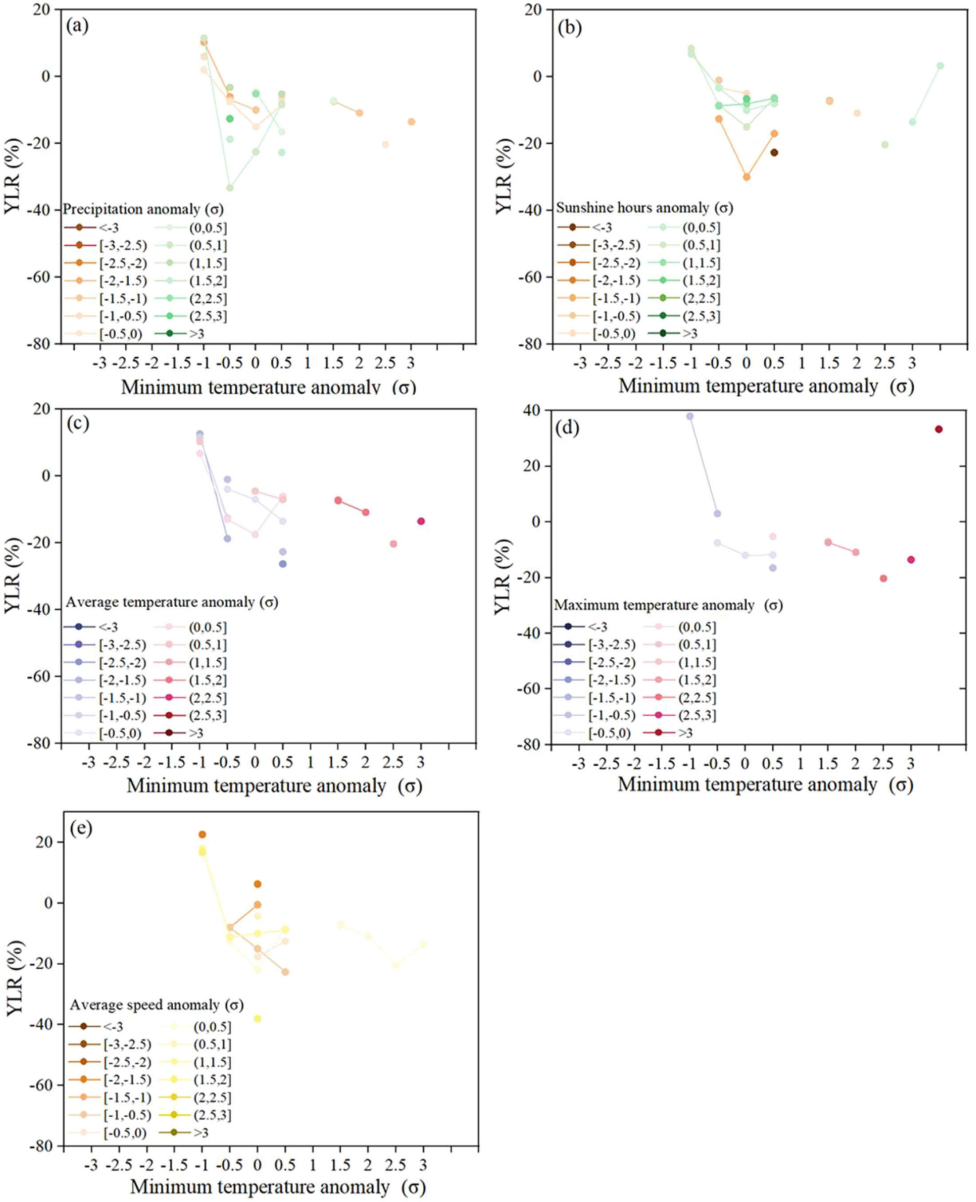
Disentangling rainfall, sunshine hours, average temperature, maximum temperature and wind speed limitation effects from minimum temperature. The minimum temperature versus yield loss ratio (YLR), binned by rainfall **(A)**, sunshine hours **(B)**, average temperature **(C)**, maximum temperature **(D)** and wind speed **(E)**. Each circle represents the YLR within each interval of rainfall, sunshine hours, average temperature, maximum temperature and wind speed anomaly.

In order to distinguish the contribution of minimum temperature on yield reduction from that of rainfall, sunshine hours, average temperature, maximum temperature and wind speed, we disentangled the independent effects of these six meteorological factors in limiting the yield reduction by exploiting the fact that each factor varies independently across different bins (**Figure 5**). In this study, we assumed that if extreme low temperature was the key limiting factor for the growth of winter wheat, the yield reduction caused by extreme low temperature would be significantly greater regardless of the fluctuations in rainfall, sunshine hours, average temperature, maximum temperature and wind speed; On the contrary, if extreme low temperature was not the key limiting factor, its inhibitory effect on yield would not result in significant yield losses due to the changes of other environmental factors. However, when looking at the variation in YLR across rainfall (**Figure 5A**), sunshine hours (**Figure 5B**), average temperature (**Figure 5C**), maximum temperature (**Figure 5D**) and wind speed (**Figure 5E**) defined in this study, the minimum temperature consistently reduced the yield in the range of rainfall, sunshine hours, average temperature, maximum temperature and wind speed. This indicates that without the coupling effects of minimum temperature-rainfall, minimum temperature-sunshine hours, minimum temperature-average temperature, minimum temperature-maximum temperature and minimum temperature-wind speed, extreme rainfall, sunshine hours, high temperature or extreme wind speed alone was insufficient to drive substantial yield decline.

### 3.4 Correlation analysis between extreme climate and winter wheat yield components

**Figure 6:**
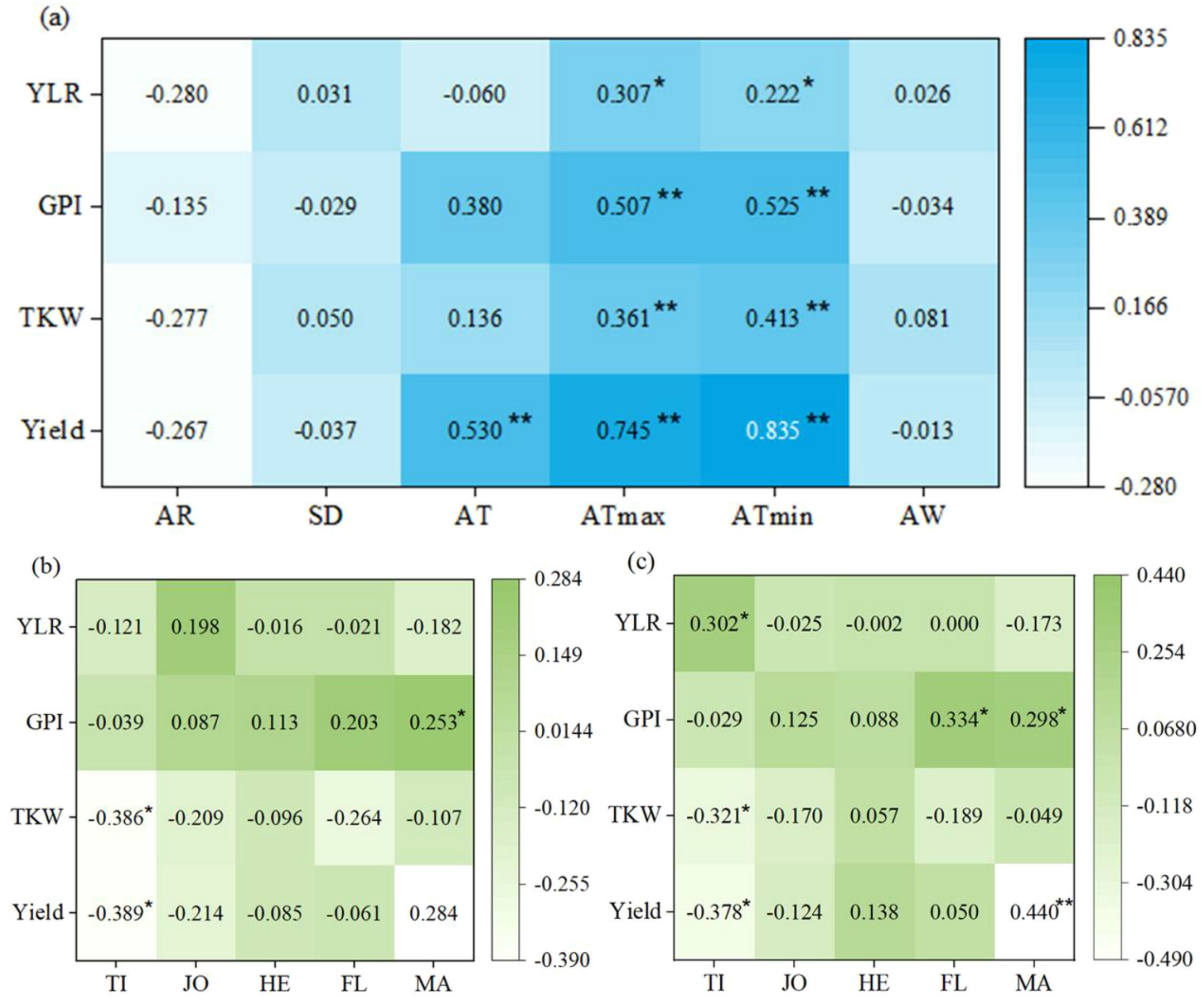
Heat map of the correlations between extreme climate and winter wheat yield components. The corresponding value of the middle heat map is the Spearman correlation coefficient r, the legend on the right is the color distinction of different r values; r<0 indicates a negative correlation, r>0 indicates a positive correlation, ‘*’ and ‘**’ represent P< 0.05 and P< 0.01 after FDR correction, respectively. **(a)**, the correlation between meteorological factors and yield components; **(b)**, the correlation between the average maximum temperature during different growth stages and yield components; **(c)**, the correlation between the average minimum temperature during different growth stages and yield components. TLR, yield loss rate; GPI, number of grains per spike; TKW, 1000-grain weight; AR, precipitation; SD, sunshine hours; AT, average temperature; ATmax, average maximum temperature; ATmin, average minimum temperature; AW, average wind speed. TI, tillering; JD, jointing; HE, heading; FL, flowing; MA, maturity.

In order to better reveal the relationship between various climate factors and yield components, Spearman correlation heatmap (**Figure 6A**) was used. The results showed that yield loss ratio (YLR) is significantly negative correlated with the average maximum temperature (r=-0.255, P<0.05) and average minimum temperature (r=-0.261, P<0.05), indicating that both high and low temperatures can exacerbate yield losses. In addition, the average maximum temperature (r=0.745) and the average minimum temperature (r=0.835) exhibited high significant positive correlation (P<0.01) with yield, 1000-grain weight (r=0.361, 0.413) and grain number per spike (r=0.507, 0.525), indicating that moderate temperature rise is beneficial for small flower differentiation, fertilization and grain filling processes. These improvements further increase the grain number per spike and 1000-grain weight, thereby increasing overall yield. The average temperature is also significantly positively correlated with the yield (r=0.530, p<0.01), indicating that temperature is the core climatic factor regulating winter wheat yield. However, precipitation, sunshine hours, and average wind speed have a certain inhibitory effect on yield and yield components, but the effect is not significant (p>0.05).

Further analysis of the correlation between the average maximum temperature during different growth stages and yield components (**Figure 6B**). The results showed that the average maximum temperature during the jointing period is weakly positive correlation with the YLR (r=0.198, P>0.05), indicating that high temperatures during the jointing period would slightly exacerbate yield loss. Compared with other, the grain number per spike is strongly positively correlated with the average maximum temperature during flowering stage (r=0.113, P>0.05) and maturity stage (r=0.203, P>0.05), indicating that moderate temperature rise during flowering facilitate pollen fertilization, while appropriate temperatures during maturity promote the translocation of photosynthetic assimilates to grains, thereby increasing the grain number per spike Additionally, the 1000-grain weight (r=-0.368, P<0.05) and yield (r=-0.389, P<0 05) are significantly negatively correlated with the average maximum temperature during the tillering stage, indicating that high temperatures during the tillering stage can damage the development of crop source organs, leading to insufficient material supply for subsequent grain filling, a significant reduction in 1000-grain weight, and ultimately a significant decrease in yield.

The correlation between the average minimum temperature at different growth stages and yield components (**Figure 6C**) showed that the YLR only significantly negative correlated with the average minimum temperature during the tillering stage (r=-0.302, P<0.05), indicating that low temperature during the tillering stage would significantly exacerbate yield loss, while the correlation with the jointing stage, heading stage, flowering stage and maturity stage was extremely weak and not statistically significant (P>0.05). Additionally, the average minimum temperature during the tillering stage is significantly negatively correlated with 1000-grain weight (r=-0.321, P<0.05) and yield (r=-0.378, P<0.05), indicating that low temperatures during the tillering disrupt the development of source organs, leading to insufficient material supply for subsequent grain filling, a significant decrease in 1000-grain weight, and ultimately a significant decrease in yield. Furthermore, the grain number per spike is significantly positively correlated with the average minimum temperature during the flowering stage (r=0.334, P<0.05) and maturity stage (r=0.298, P<0.05), respectively. It can be seen that a gradual increase in the average minimum temperature during flowering and maturity promotes pollen fertilization, fruiting, and the translocation of photosynthetic assimilates to grains, thereby significantly increasing the grain number per spike. Yield is significantly positively correlated with the average minimum temperature during maturity (r=0.440, P<0.01), confirming that suitable temperature rise during maturity is conducive to yield improvement. Meanwhile, a weak positive correlation was observed between yield and the average minimum temperature during the heading stage (r=0.138, P>0.05), which may be attributed to the fact that the average minimum temperature at heading stage has risen to the appropriate temperature range for winter wheat, exerting a slight promoting effect on yield formation.

### 3.5 Multivariate analysis of factor importance

To formally assess the relative importance of each meteorological factor in limiting yield, we conducted a dominance analysis based on a multiple linear regression model with YLR as the dependent variable and the six standardized climate anomalies as predictors. The model explained 58% of the variance in YLR (adjusted R² = 0.54). Dominance analysis revealed that average minimum temperature was the most important predictor, contributing 41% of the explained variance (generalized dominance weight = 0.41), followed by wind speed (22%), average temperature (15%), precipitation (12%), average maximum temperature (6%), and sunshine hours (4%). These results confirm that minimum temperature is the dominant meteorological limiting factor for winter wheat yield in Shihezi.

## 4 Discussion

### 4.1 Differential impact of various meteorological factors on yield

The actual yield of winter wheat ranges from 3480 to 9350 kg·hm^-2^ in Shihezi during 1980-2024, showing an overall increasing trend. The trend is mainly attributed to the increased fertilizer application, cultivation of excellent varieties and construction of irrigation facilities (Gleiser et al., 2021). In order to facilitate the analysis of meteorological factors affecting yield changes, six commonly used meteorological factors are selected in this study. The results showed that the magnitude of the impact of these meteorological factors on yield variation followed the order: average minimum temperature > wind speed > average temperature > precipitation > average maximum temperature > sunshine hours, reflecting that winter wheat yield in Shihezi is mainly limited by low temperature to some extent. Consistent with this finding, Xu (2009) also reported that the average minimum temperature is the core climatic factor affecting the growth and yield of winter wheat in the Shihezi area. Gao et al. (2025) found that significant climate variability during the growth period of winter wheat in Xinjiang, with precipitation, average minimum temperature and average maximum temperature being the core climate variables affecting yield. However, the present study revealed that temperature changes at different growth stages exert differential effects on winter wheat, reflecting discrepancies in research results between site-specific and provincial scales. Tao et al. (2008) also found that the climate yield relationship at the national or provincial level cannot be applied to the municipal level, highlighting the complexity of climate impacts on yield.

Specifically, regarding precipitation, the positive YLR were observed under moderate drought conditions of -1.0 σ ~ 0 σ, indicating that winter wheat in this region exhibits moderate drought tolerance. This is associated with the local physiological adaptation mechanisms developed through long-term acclimation to arid environments. However, when the precipitation anomaly is below -1.0 σ or above 1.0 σ, the YLR significantly increases, indicating that water stress (either drought or excessive humidity) exacerbates the yield reduction caused by extreme low temperatures (Li et al., 2014). This result differs from the humid areas in southeastern regions of China, where rainfed agriculture dominates and excessive precipitation is prone to waterlogging and yield reduction (Liu et al., 2022). While the annual precipitation in Shihezi is only 117.3-368.5 mm, and water is not the primary limiting factor for winter wheat growth due to the support of irrigation facilities. Moderate drought is actually conducive to root development and nutrient accumulation (Chen et al., 2012). For sunshine hours, although the yield loss caused by short sunshine is higher than that caused by long sunshine, the maximum loss rate does not exceed -7%, which is far lower than the impact of extreme low temperatures. This may be due to the sufficient annual sunshine base of 2031.1-3220.7 h in Shihezi. Short sunshine stress only delays the growth process without causing irreversible damage to reproductive growth. Extreme high temperatures can also lead to a reduction in winter wheat yield, but the YLR (-13.54%) when the average maximum temperature anomaly reaches 2.0 σ is significantly lower than that of extreme low temperatures. High temperatures during different growth stages have differential effects. High temperatures during the tillering stage can impair the development of crop source organs, leading to insufficient material supply for subsequent grain filling, a significant reduction in 1000-grain weight, and ultimately a marked decrease in yield (Smith et al., 2023). In addition, the average wind speed only induced significant yield reduction within the anomaly range of 0.5 σ ~ 2 σ, with a maximum loss rate of -31.23%. However, there is no extreme strong winds (anomaly ≥ 2.0 σ) were recorded in the region, so the actual threat of wind speed to yield is less than that of extreme low temperatures. This may be related to the relatively few strong wind days during the winter wheat growing season in Shihezi.

### 4.2 The main limiting impact of extreme low temperature on winter wheat yield

The Shihezi region is located in a temperate continental climate zone, where winter wheat overwintering for 5–6 months without stable snow cover, making its growth stages extremely sensitive to temperature fluctuations. This study found that the average minimum temperature is significantly positively correlated with the YLR (r=-0.261, P<0.05), and it mainly inhibit yield formation by reducing the 1000-grain weight (r=-0.321, P<0.05) at tillering stage. This is consistent with the research conclusions on the impact mechanism of low temperature disasters in northern wheat-growing regions (Zhang et al., 2021). Compared with the average temperature and the average maximum temperature, the interannual CV of the average minimum temperature is lower (0.12~0.18), but extreme low temperatures caused more severe yield damage. One possible reason is that nighttime low temperature not only directly damages crop cell structure, but also seriously affect the accumulation of photosynthetic assimilates in functional leaves (Marcellos and Single, 1979), and this effect is difficult to alleviate through self-regulation during the critical period of reproductive growth (Zhang W. J. et al., 2019). A comparison with studies in the middle and lower reaches of the Yangtze River—where excessive precipitation is the main yield-limiting factor (Liu et al., 2022), further highlights the regional specificity of meteorological limiting factors. In the arid and low-rainfall climate of Shihezi, water is not the primary limiting factor due to irrigation support; instead, extreme low temperatures impose more prominent constraints on winter wheat overwintering survival, growth, and development. This regional difference provides a reference for formulating differentiated disaster prevention and mitigation strategies across different climate zones. In general, extreme low temperature is the key meteorological factor that limits winter wheat yield in the Shihezi region.

### 4.3 Future research directions

This study indicates that low temperature is the most critical meteorological limiting factor affecting winter wheat yield in Shihezi, compared with precipitation, sunshine duration, and average wind speed. Based on this research result, targeted measures can be taken to mitigate the impacts of extreme low temperatures, such as breeding cold-resistant varieties, optimizing sowing dates to avoid critical low-temperature periods, and adopting film mulching for cold and moisture preservation during the overwintering stage. On the other hand, this study relies on correlation analysis of historical data, which can only reveal past variation patterns and is insufficient to accurately predict the yield response of winter wheat under future climate change scenarios. Future research can integrate crop models to simulate changes in the frequency and intensity of extreme low-temperature events under different future climate scenarios, thereby systematically assessing winter wheat yield risks. Additionally, the research scope can be expanded to the entire economic belt on the northern slope of the Tianshan Mountains to explore the spatial differentiation patterns of extreme low-temperature impacts on winter wheat yield, providing scientific references for regional-scale food security planning.

### 4.4 Limitations and future directions

This study has several limitations. First, while we used robust statistical methods to detrend yield data and isolate climate signals, correlation-based approaches cannot prove causation. The identified relationships are consistent with known physiological mechanisms, but experimental validation (e.g., controlled environment studies) would strengthen causal inference. Second, our analysis is based on a single location; if the scope of analysis is expanded to the broader Tianshan North Slope Economic Belt, it will reveal the spatial distribution pattern of cold risks, thereby providing a basis for regional-level food security planning. Third, the relatively small number of extreme cold years (n = 3) in the 45-year record means that the -43.6% YLR estimate, while robust to bootstrapping, is based on limited samples. Continued monitoring is needed to track future events. Finally, historical analysis cannot predict future risks under climate change. Integrating crop models (e.g., CERES-Wheat, APSIM) with future climate scenarios would allow for forward-looking assessments of how the frequency and intensity of extreme low-temperature events may change, and what that implies for wheat production.

## 5 Conclusions

In this study, we proved that, compared with precipitation, sunshine hours and average wind speed, extreme low temperature is the key meteorological limiting factor leading to a 43.58% reduction on winter wheat yield in Shihezi, far exceeding that of other meteorological stresses such as extreme wet and extreme high temperature. The inhibitory effect of extreme low temperature on yield has growth stage-specificity by mainly affecting the development of crop source organs during the tillering stages, significantly reducing the 1000-grain weight, and ultimately leading to yield decline. Meteorological factors such as precipitation, sunshine hours, and wind speed have a certain impact on winter wheat yield, but the intensity of their effects is lower than that of extreme low temperatures. Therefore, clarifying the potential impacts of climate change on winter wheat production in the study area, we can closely combine the findings with meteorological forecasting techniques and transform them into targeted meteorological services for winter wheat production. Effective field management measures can then be taken to mitigate the impact of adverse meteorological conditions on wheat production, providing meteorological guarantees for increasing wheat yield and income. In addition, historical data could only reveal the correlation between past climate and crop production, and it is difficult to predict future dynamic changes. Crop models simulation with potential future climate scenarios will effectively reveal the potential risks of climate change to crop production.

## Data Availability

The datasets presented in this article are not readily available because This original dataset is classified as confidential. Requests to access the datasets should be directed to Shuai Zhang, 1048022597@qq.com.
